# Development and validation of chemometric-assisted spectrophotometric models for efficient quantitation of a binary mixture of supportive treatments in COVID-19 in the presence of its toxic impurities: a comparative study for eco-friendly assessment

**DOI:** 10.1186/s13065-023-01089-9

**Published:** 2023-12-07

**Authors:** Heidi R. Abd El-Hadi, Maya S. Eissa, Hala E. Zaazaa, Basma M. Eltanany

**Affiliations:** 1 Faculty of Pharmacy, Pharmaceutical Chemistry Department, Egyptian Russian University, Badr City, Cairo Egypt; 2 Faculty of Pharmacy, Analytical Chemistry Department, Cairo University, Kasr El-Aini Street, Cairo, 11562 Egypt

**Keywords:** Chemometric models, COVID-19 supportive treatment, Paracetamol, Hyoscine *N*-butyl bromide, Greenness assessment

## Abstract

The use of sustainable solvents has increased significantly in recent years due to advancements in green analytical methods. The number of impurities in the drug substance determines how safe the finished product is. Therefore, during the whole medication planning process, contaminants need to be closely watched. Using chemometric models, the concentrations of hyoscine *N*-butyl bromide (HYO) and paracetamol (PAR) were determined in the presence of three PAR impurities [P-nitrophenol (PNP), P-aminophenol (PAP), and P-chloroacetanilide (PCA), as well as DL-tropic acid (TRO) as a HYO impurity]. It was possible to isolate and measure these dangerous impurities. Fever and spasms associated with COVID-19 are reported to be considerably reduced when PAR and HYO are taken together. Artificial neural networks, principal component regression, multivariate curve resolution-alternating least squares, and partial least squares are the four chemometric-assisted spectrophotometric models that were created and verified. All of the proposed methods’ quantitative analytical potency was assessed using recoveries%, root mean square error of prediction, and standard error of prediction. For PAR, HYO, PNP, PCA, TRO, and PAP, respectively, the indicated approaches were used in the ranges of 4.00–8.00, 16.00–24.00, 1.00–5.00, 0.40–0.80, 4.00–12.00, and 2.00–6.00 µg/mL. They are able to get around difficulties like collinearity and spectral overlaps. After statistical testing, there was no discernible difference between the recommended methods and the published one. The degree of greenness of the established models was evaluated using three different green assessment methods. In the presence of their harmful impurities, PAR and HYO could be identified using the recommended methods.

**Supplementary Information:**

The online version contains supplementary material available at 10.1186/s13065-023-01089-9.

## Introduction

The terms "green analytical chemistry" (GAC), "environmental impact," "sustainable development," and "waste reduction" are frequently used interchangeably [1]. The Eco-Scale, Analytical GREEnness metric (AGREE), and Green Analytical Procedure Index (GAPI) are the methods available for assessing how environmentally friendly an analytical approach is [2]. Based on penalty points, the founded techniques eco-scaling was assessed, and deducted from a base of 100. From samples taken until assessment in the end, the trustworthy methodologies AGREE and GAPI are capable of supplying an extensive ecological evaluation of the entire analytic process [3]. Each solvent is therefore marked with a label that includes a description and a color code, such as "recommended" being indicated in green and "dangerous" by red [4].

SARS-CoV-2, a newly identified coronavirus that was first observed in China in December 2019, is the problem causing of the coronavirus disease 2019 (COVID-19) [5–8]. Increasing of body temperature, coughing, breathlessness, dry mouth, nasal congestion, migraines, diarrhea, spasm, nausea, and vomiting are the most typical COVID-19 symptoms [9].

Paracetamol (PAR), also known by its chemical name 4′ hydroxyacetanilide, is a common antipyretic and analgesic as shown in Additional file 1: Fig. S1 [10–12]. It is listed as a recognized drug in the British Pharmacopoeia (BP) and American Pharmacopoeia (USP) [13–15]. Recently, PAR was referred to as the main analgesic and antipyretic for the treatment of COVID-19 symptoms [9]. An anti-cholinergic quaternary ammonium compound called hyoscine *N*-butylbromide (HYO) is shown in Additional file 1: Fig. S1 [16]. It functions by calming the intestine and stomach muscles. Cramps, pain, bloating, and discomfort are reduced as unanticipated muscle spasms are avoided by HYO [17]. As a result, HYO was recently suggested as a treatment for COVID-19 symptoms like spam, nausea, and vomiting [18–20]. Recently, the combination of PAR and HYO was recommended by WHO as a treatment for COVID-19 symptoms such as fever, sore throat, headache, spasms, nausea and vomiting [18, 21, 22]. Each Buscopan Plus^®^ tablet contains 500.00 mg of PAR and 10.00 mg of HYO, according to the manufacturer's instructions [23]. These tablets can ease the cramps and abdominal pain brought on by COVID-19.

In B.P. opinion, the following impurities, such as P-aminophenol (PAP), P-nitrophenol (PNP), and P-chloroacetanilide (PCA), may be present during the PAR manufacturing process (Additional file 1: Fig. S1) [14]. These impurities might be involved in the synthesis process or produced as degradation byproducts during storage. PAP is categorized as impurity "K" for PAR and is a nephrotoxic impurity whose concentration must not be higher than 50.00 ppm [24]. PNP, which is regarded as an impurity "F" for PAR and has a limit of 500.00 ppm, can cause methemoglobinemia [25]. Since it harms the skin and eyes, PAC is a PAR impurity "J" that should not be present in concentrations higher than 10.00 ppm. It may have a toxic side effect on the liver and kidneys [26]. DL-tropic acid (TRO) is categorized as impurity "B" for HYO causes skin, eye and respiratory irritation so its level must be controlled and not excessed 2000.00 ppm [14]. Therefore, developing a new analytical approach for assessing the two main compounds in the presence of specific toxic impurities and further assessing these impurities is a demanding and challenging task.

Spectrophotometric evaluation of this mixture in its pharmaceutical formulation for the concurrent assessment of PAR and HYO was found in the literature through a variety of sources [27, 28]. The quantitative assessment of this mixture has also been indicated using a variety of chromatographic techniques [23, 29, 30]. There is only one green chromatographic method was reported for estimation of the PAR and HYO in a binary mixture in presence of their toxic impurities [17].

Chemometry is thought to be more effective than one-variate calibration techniques for assessing complicated mixtures [31]. It has a wide range of uses and can extract the most data from datasets that are provided [32–34]. The concurrent existence of several spectral wavelengths, which offers greater accuracy than a specific wavelength is a key component of the multivariate calibration technique that can identify highly overlapping spectra [35]. Through the use of a multivariate model, this method can be utilized to quickly forecast analytes concentrations by analyzing spectra from unknown substances [36, 37]. Additionally, multivariate calibrations are thought to be an effective tool for spectral analysis because they can use a variety of spectral intensities, each of which has a huge impact on precision [38, 39].

The work aims to develop the first green chemometric methods to use GAC for quantitative and qualitative analysis of PAR and HYO in the presence of toxic impurities. Modern chemometric techniques for multicomponent matrix resolution including partial least squares (PLS), classical principal component regression (PCR), artificial neural networks (ANN), and multivariate curve resolution-alternating least squares (MCR-ALS) were used for determination our binary mixture in the presence its toxic impurities. In terms of green assessment, the developed methods were compared with a published approach utilizing eco-scaling, AGREE, and GAPI [17]. There were no appreciable differences discovered when the suggested procedures were statistically examined using the published HPLC technique [29].

## Experimental

### Equipment and software

The UV–visible spectrophotometer JASCO dual beam model V-630 (Tokyo, Japan) was utilized with the spectra II manager provided in the package. The scanning rate was 1000 nm/min, and the spectral slit width was 2 nm. MATLAB^®^ 8.3.0.532 (R2014a), PLS Toolbox (version 2.1), MCR-ALS Toolbox [40], and Neural Network Toolbox were used to implement all chemometric approaches (Math Works, United States).

### Chemicals and reagents

Delta Pharm for Pharmaceutical Industry generously contributed PAR and HYO (Cairo, Egypt). Their purity was determined to be 99.25% ± 0.792 for PAR and 99.73% ± 1.012 for HYO by official measurements [14]. PAP, PNP, PCA and TRO were given by Sigma Aldrich (Darmstadt, Germany). Analytical grade methanol was bought from El-Nasr Pharmaceutical Chemical Company in Cairo, Egypt. The Buscopan Plus^®^ tablets were made by Sanofi Company. Each tablet active ingredients are listed as 500.00 mg of PAR and 10.00 mg of HYO.

### Preparation of standard solutions

Stock standard solutions of PAR, HYO, PNP, PCA, TRO, and PAP were made in methanol at a concentration of 1.00 mg/mL each. The corresponding stock standard solutions of PAR, HYO, PNP, TRO, and PAP in methanol were used to create working standard solutions (100.00 µg/mL). The corresponding stock standard solutions of PCA in methanol were used to create the working standard solutions (5.00 µg/mL).

### Procedures

#### Zero-order absorption spectra

The PAR, HYO, PNP, PCA, TRO, and PAP absorption spectra were acquired spanning the wavelength range of 200.0–400.0 nm. For further data analysis, the spectral data points with a wavelength range of 230.0–266.0 nm were imported into Matlab^®^. The selected wavelength range show high interference between the developed drug and its impurities.

### Calibration and validation sets

Using the five-level, six-factor experimental design and five concentration levels labeled from + 2 to − 2 for each of the two drugs and its impurities under consideration, calibration and validation datasets were produced [41]. For the calibration and validation sets, the concentration ranges for PAR, HYO, PNP, PCA, TRO, and PAP were respectively (4.00–8.00 µg/mL), (16.00–24.00 µg/mL), (1.00–5.00 µg/mL), (0.40–0.80 µg/mL), (4.00–12.00 µg/mL), and (2.00–6.00 µg/mL). Transferring various concentrations of PAR, HYO, PNP, PCA, TRO, and PAP from their corresponding working solutions to a series of 10-mL volumetric flasks resulted in the creation of 25 samples that were mixtures of PAR, HYO, PNP, PCA, TRO, and PAP in different ratios. To assess the predictive power of each calibration model, eight samples were randomly selected for the cross-validation set, and the remaining samples were utilized to create the regression model. The spectral data of the calibration and validation sets were scanned at intervals of 0.1 nm. After that, the data was imported into MATLAB^®^ then processed further using PLS, PCR, ANN, and MCR-ALS multivariate calibration models. Every aspect of the approach was reviewed and enhanced.

### Regression calibration optimization

#### Principal component regression and partial least squares

The number of latent variables (LVs) in the established calibration models was modified using mean-centered data and leave-one-out cross-validation. Eight LVs were the optimum number to obtain the lowest root mean square calibration error (RMSEC).

#### Artificial neural networks

The feed-forward model training process enhanced the ANN calibration method. There were 361 and 6 neurons, respectively, in the input and output layers. Additionally, efforts were made to maximize the number of neurons in the hidden layer. The purelin-purelin transfer function was used to select 6 neurons from the hidden layer. This function is frequently used for linear processes. Furthermore, it was found that the optimum number of epochs is 100.

#### Multivariate curve resolution-alternating least squares

The non-negativity constraints, which were obtained using the fast non-negativity constrained least squares algorithm (fnnl), were implemented to both spectral and concentrations profiles to obtain the best features with the fewest iterations. These constraints were the most important optimization factor in MCR-ALS calibration.

### Greenness assessment

It was critical to investigate each analytical method environmental impact to determine whether it corresponded to the green chemistry theory. Different methods can be used to evaluate how green something is [42]. Four main criteria were used to evaluate how environmentally friendly analytical techniques were: high energy consumption, high waste production, excessive chemical use, and its risks, and the use of a lot of chemicals [4, 43]. Three analytical tools were used to evaluate the suggested technique greenness and cost-effectiveness. They were as follows:

### Green analytical procedure index

It is a tool that offers extensive knowledge of fifteen analytical technique areas, each represented by five pentagrams [4]. According to the GAPI color scheme, red denotes a significant environmental risk, yellow denotes a lower ecological tolerance, and green denotes a higher ecological tolerance [44].

### Analytical GREEnness metric

General guidelines like inclusivity, input flexibility, clarity, and yield clarity were used in the development of the AGREE tool [42]. Twelve segments make up the automatically generated pictogram, with the ability to change the width of each segment according to its importance [4]. The colors used for each section range from deep green to deep red [2]. The final score is displayed in the circular pictogram center. The final AGREE score is a decimal that ranges from zero to one [4].

### Analytical eco-scale

Utilizing an analytical method known as the "eco-scale," it is possible to compare options and select the one that is the greenest [42]. 100 penalty points serve as the starting point. Each analytical technique parameter's penalty points are determined and are downgraded from 100. The more points, the more environmentally and economically responsible the analytical method [45, 46].

### Pharmaceutical preparation analysis

In a neat mortar, ten tablets were weighed and a fine powder was made from them. A part of the weighed smashed powder was put into a 100 mL volumetric flask containing 60 mL of methanol to create a solution that contained 5000.00 µg/mL PAR and 100.00 µg/mL HYO. To complete the volume, methanol was added after 30 min of sonication. The filtration process was then done then transferring 2.5 mL from the stock solutions into a 100 mL volumetric flask allowed for the creation of a working solution with concentrations of 125.00 µg/mL for PAR and 2.50 µg/mL for HYO. Aliquot concentrations of this solution were transferred into 25 mL volumetric flasks (5.00 µg/mL for PAR and 0.10 µg/mL for HYO). The resulting solution was spiked with 16.00 µg/mL HYO and methanol was used to bring the volume up to the desired level. It was necessary to raise HYO concentration because it was extremely low in the mixture. This was accomplished by adding a predetermined amount of standard HYO to be examined. Before determining the claimed drug concentration, we first removed the HYO concentration. Following the aforementioned methods, each compound concentration was determined using the associated regression equations. Analysis of dosage form solutions was done using the suggested models, as indicated under linearity. To calculate the concentrations of the substances under inquiry, regression equations were constructed.

## Results and discussion

During the synthesis process or as a result of improper pharmaceutical compound storage, impurities and degradation products are generated. PAR can degrade easily and contain impurities such as PNP, PCA and PAP while HYO can contain impurity such as TRO. There have been many published spectrophotometric approaches for assessing PAR and HYO in dosage forms, but as of now, no chemometric methods have been documented for determining our binary mixture in the presence of toxicities. Multivariate calibrations are employed in quality control laboratories not only for pharmaceutical testing but also for the identification of impurities in drug formulations and bulk drugs [2].

**Fig. 1:**
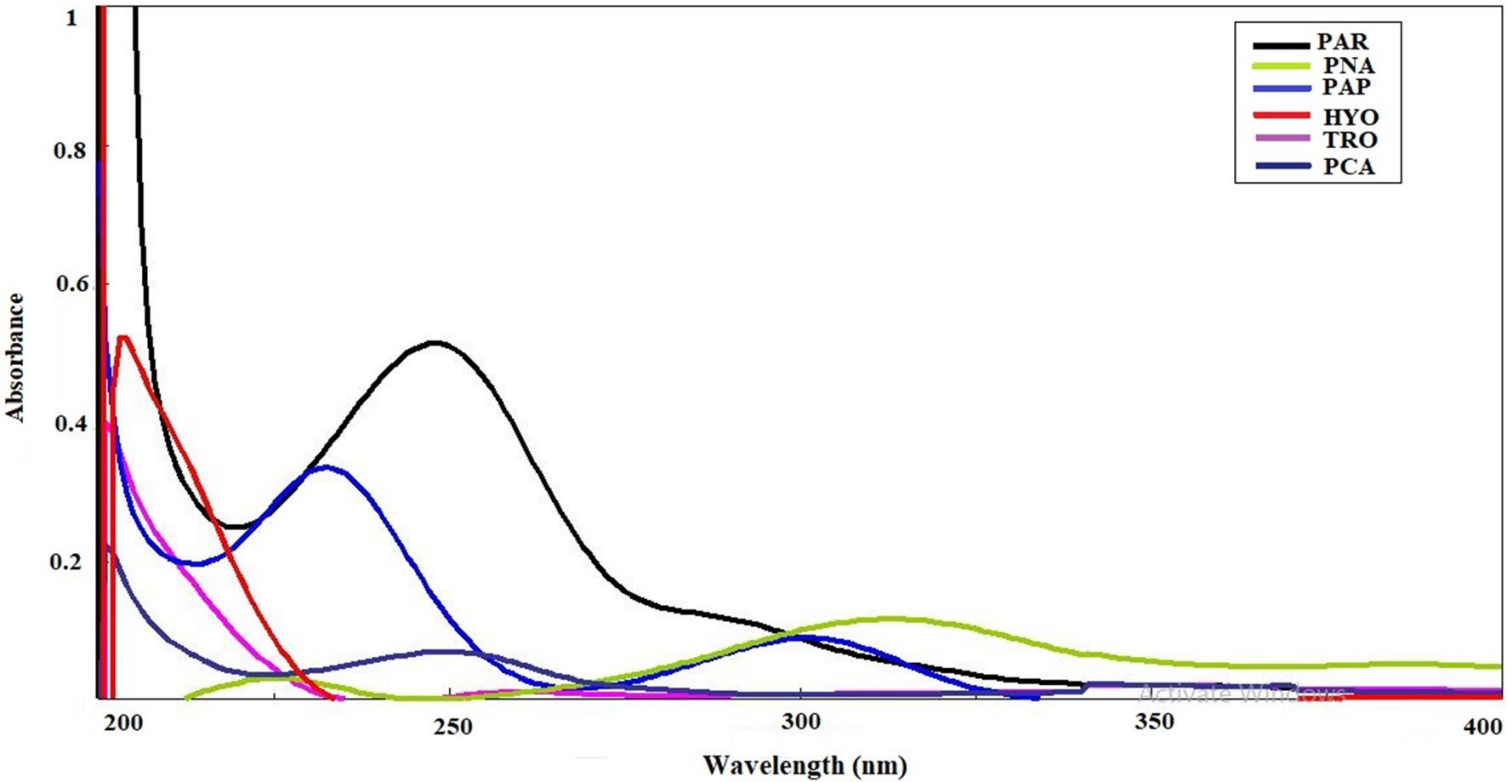
UV-spectra of paracetamol (6.00 µg/mL), hyoscine butylbromide (20.00 µg/mL), p-aminophenol (5.00 µg/mL), p-nitrophenol (2.00 µg/mL), p-chloractanilide (0.60 µg/mL) and tropic acid (8.00 µg/mL) product using methanol as solvent

In this study, substantially overlapping drug spectra were resolved using multivariate data processing (Fig. 1).

Four multivariate methodologies were employed in the investigation of PAR, HYO, PNP, PCA, TRO, and PAP. A thorough experimental design of the calibration set composition is necessary for the multivariate calibration to yield the perfect guess. A multilevel multifactor design was applied to create the samples, with the training set consisting of 17 samples and the validation set consisting of the remaining 8 samples (Table 1). The best outcomes came from scanning a range of spectra between 230.0 and 266.0 nm at 0.1 nm intervals. The established models were calibrated and assessed using 361 experimental points. Calibration models were proposed, examined, and utilized to forecast unknown samples.

**Table 1:** Concentrations of PAR, HYO, PNP, PCA, TRO and PAP in the calibration and validation sets for the multivariate calibrations

Mix no.	PAR	HYO	PNP	PCA	TRO	PAP
1	6.00	20.00	3.00	0.60	8.00	4.00
2	6.00	16.00	1.00	0.80	6.00	6.00
3	4.00	16.00	5.00	0.50	12.00	4.00
4	4.00	24.00	2.00	0.80	8.00	3.00
**5**	**8.00**	**18.00**	**5.00**	**0.60**	**6.00**	**3.00**
6	5.00	24.00	3.00	0.50	6.00	5.00
7	8.00	20.00	2.00	0.50	10.00	6.00
**8**	**6.00**	**18.00**	**2.00**	**0.70**	**12.00**	**5.00**
9	5.00	18.00	4.00	0.80	10.00	4.00
**10**	**5.00**	**22.00**	**5.00**	**0.70**	**8.00**	**6.00**
**11**	**7.00**	**24.00**	**4.00**	**0.60**	**12.00**	**6.00**
12	8.00	22.00	3.00	0.80	12.00	2.00
13	7.00	20.00	5.00	0.80	4.00	5.00
**14**	**6.00**	**24.00**	**5.00**	**0.40**	**10.00**	**2.00**
**15**	**8.00**	**24.00**	**1.00**	**0.70**	**4.00**	**4.00**
16	8.00	16.00	4.00	0.40	8.00	5.00
**17**	**4.00**	**22.00**	**1.00**	**0.60**	**10.00**	**5.00**
18	7.0	16.00	3.00	0.70	10.00	3.00
19	4.00	20.00	4.00	0.70	6.00	2.00
20	6.00	22.00	4.00	0.50	4.00	3.00
21	7.00	22.00	2.00	0.40	6.00	4.00
**22**	**7.00**	**18.00**	**1.00**	**0.50**	**8.00**	**2.00**
23	5.00	16.00	2.00	0.60	4.00	2.00
24	4.00	18.00	3.00	0.40	4.00	6.00
25	5.00	20.00	1.00	0.40	12.00	3.00

Bolded rows are concentration of samples in the validation set

### Partial least squares and principal component regression

When developing multivariate calibration models, the two inverse least squares algorithms that are most frequently utilized are PCR and PLS [47, 48]. In the statistical study of spectra, PLS and PCR models are frequently employed to extract specific data from more general data [41]. The data concerning responses and levels are taken into account by the PLS and PCR algorithms simultaneously [49]. In this study, the proper number of components was determined by cross-validating, leave one out technique while omitting one sample at a time.

**Fig. 2:**
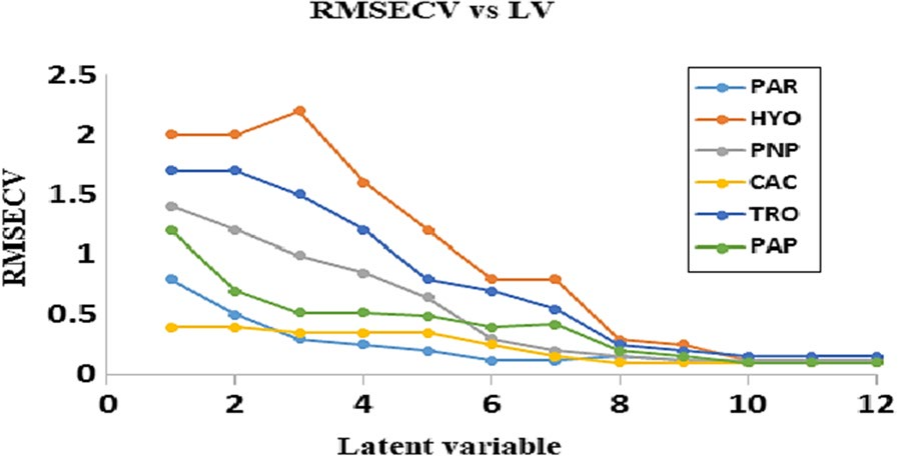
RMSECV plot of the cross-validation results of the calibration set as a function of the number of latent variables used to construct PLS calibration

**Fig. 3:**
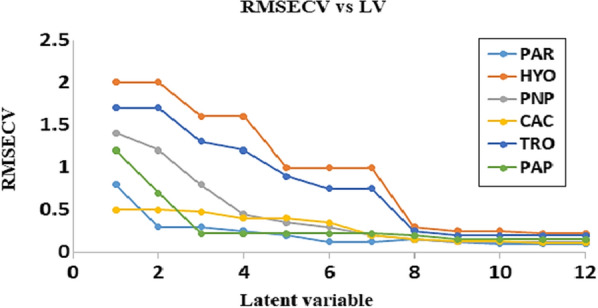
RMSEV plot of the cross-validation results of the calibration set as a function of the number of latent variables used to construct PCR calibration

The most significant prediction errors produced by the least number of LVs is the optimal number of LVs. It was determined that 8 latent variables were the ideal number in this situation (Figs. 2, 3).

### Artificial neural networks

**Fig. 4:**
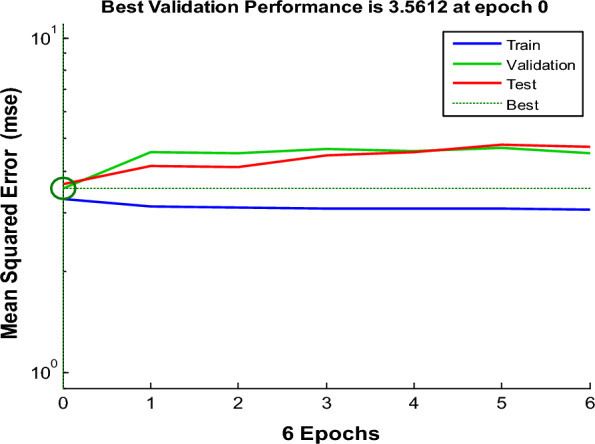
Best validation performance for the prediction of ANN model

**Fig. 5:**
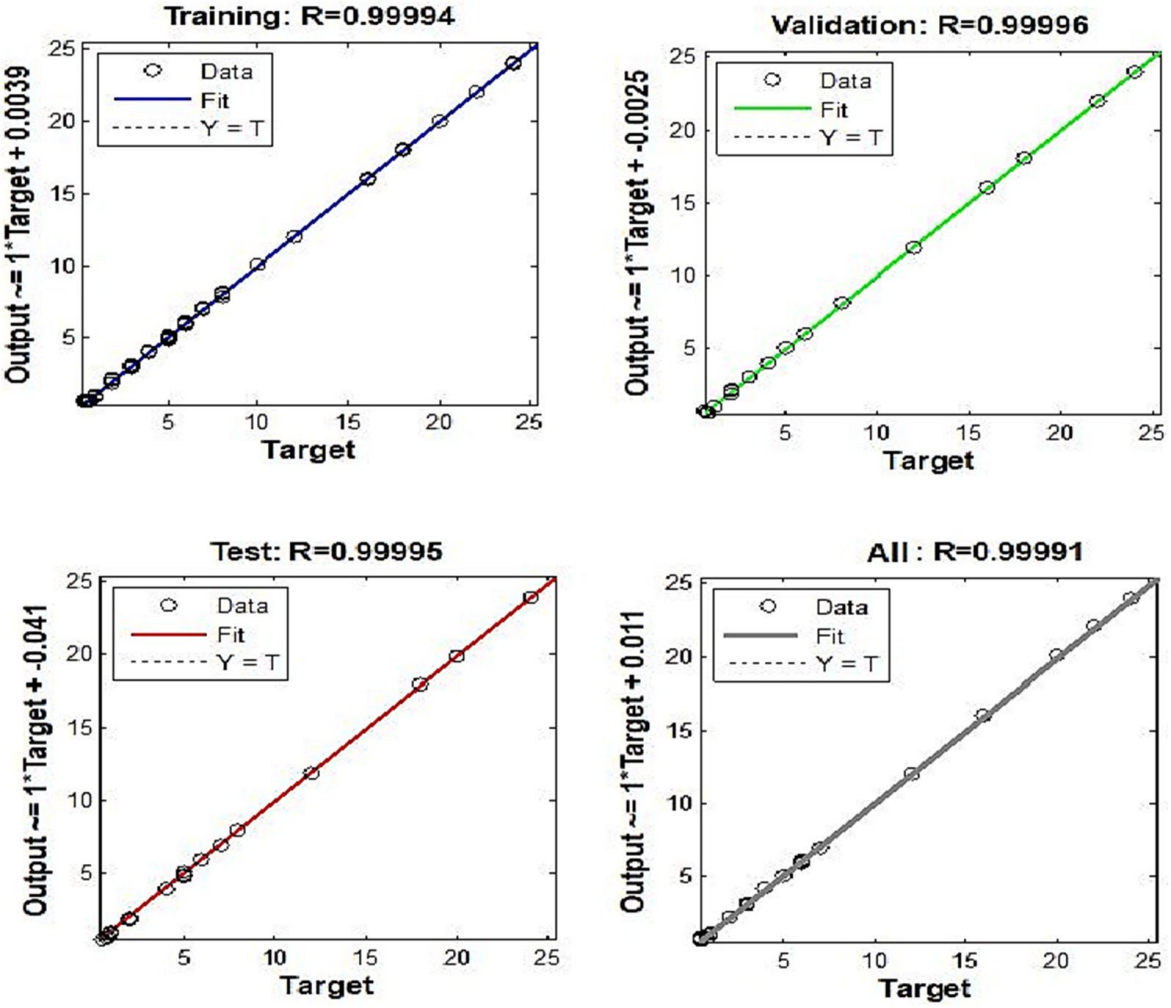
Prediction for the training, test and validation diagrams of ANN model

To identify links between inputs and outputs, ANN uses a significant number of basic, tightly connected nodes. Normally, an ANN has three layers: input, hidden, and transfer functions [50, 51]. The ANN type was utilized in this study and a feed-forward model of that type was instructed. Six neurons were utilized in the output layer, which corresponds to how many parts there were going to be assessed for each compound, and 361 neurons were applied in the input layer, which represents the number of spectral data points that were applied. Changing the number of neurons in the hidden layer should be done through trial and error. Six hidden neurons were the ideal number for a purelin-purelin transfer function and 100 epochs (Fig. 4) displays the output plot of a properly trained ANN for mean squared error (MSE) vs epochs. After epoch = 0, the MSE of training rapidly decreased. There was no overfitting, as evidenced by the similarity of the trial and validating curves and the absence of any abrupt shifts. Prediction diagrams for the chosen layers and neurons' training, test, and validation series are also displayed in (Fig. 5). A correlation coefficient value (r) that is nearer to 1 for the validation, test, and training series indicates that this model is highly effective at making predictions [52].

### Multivariate curve resolution-alternating least squares

Beer's-Lambert law's multi-wavelength extension and the bilinear model that MCR assumes are both obtained using factor analysis [53]. The recorded spectra data matrix in MCR is sorted out into spectral and concentration profile matrices for each drug in the samples, and mistakes are then detected [47, 54]. The ALS method was designed to predict concentrations from spectral characteristics repeatedly and vice versa. It is possible to reduce the number of potential solutions for data matrix decomposition by imposing restrictions such as uni-modality, closure, equality, or non-negativity.

In order to begin ALS optimization, the "easy-to-use interactive self-modeling analysis" technique was utilized to calculate the spectral-profile matrix [55]. In addition, the spectral-profile matrix was applied to compute the unconstrained least-squares solution for the concentration profile.

**Fig. 6:**
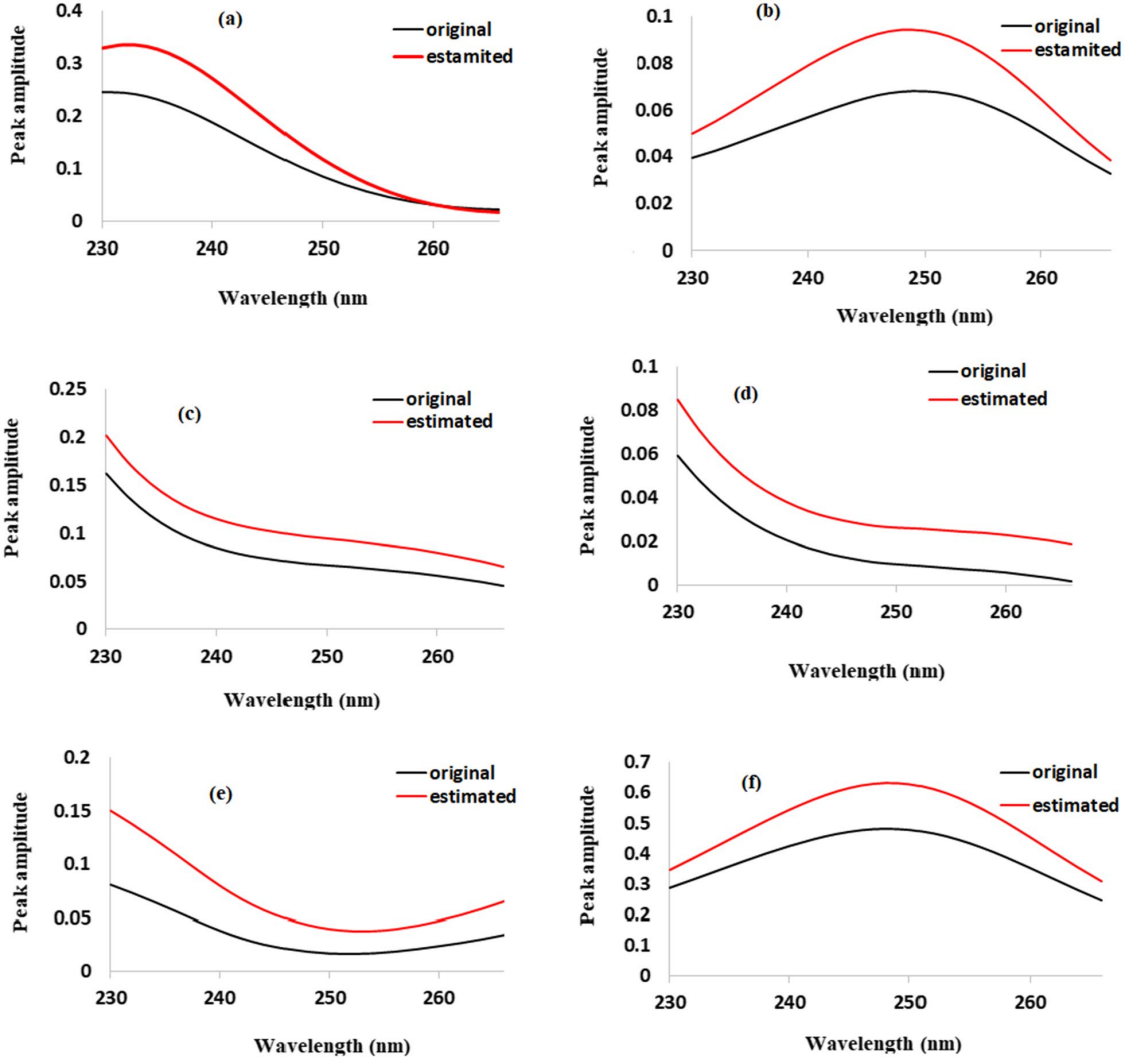
Original spectra and estimated spectra by MCR-ALS of **a** PAR, **b** HYO, **c** PNP, **d** PCA, **e** TRO, **f** PNP

In the current work, a non-negativity requirement was placed on both spectral and concentration profiles. To meet the non-negativity criterion, both spectra and concentration must equal 6. ALS effective algorithm was completed when a certain convergence threshold was met (30%). When the variance between the root mean square of "E" the residuals matrix, between numerous iterations, is less than a threshold level, the convergence is commonly stopped (typically set at 0.1%) [52]. Iterations are carried out until the desired outcome is obtained, satisfying both the predetermined convergence criteria and the predicted constraints. The convergence was broken after two repetitions. According to the computed percentages of variance (R^2^) and lack of fit (% lof), which were 100 and 0.0237, respectively, they were both very good and supported the efficacy of the developed MCR-ALS method. This method could be applied to evaluate the spectral profile of components. It is evident that the calculated and actual spectra are similar (Fig. 6).

The recovery values, mean recovery values, and RSD% are displayed in (Table 2). Utilizing the ICH criteria, the recently proposed procedures were verified [56]. Validation parameters for the validation sets were summarized in (Table 3). The proposed models additionally estimated the root mean square error of prediction (RMSEP) and RMSEC for each component.

**Table 2:** Prediction of validation set samples using the proposed chemometric methods; PLS and PCR (a) ANN and MCR-ALS (b)

(a) Prediction of validation set samples using PLS and PCR models																	
Concentration (µg/mL)						PLS						PCR					
PAR	HYO	PNP	PCA	TRO	PAP	PAR	HYO	PNP	PCA	TRO	PAP	PAR	HYO	PNP	PCA	TRO	PAP
8.00	18.00	5.00	0.60	6.00	3.00	100.05	95.30	100.61	97.59	103.11	101.37	98.08	97.85	102.47	101.12	99.12	101.72
6.00	18.00	2.00	0.70	12.00	5.00	98.75	100.40	98.14	100.43	99.63	98.11	99.28	100.72	99.09	102.86	100.15	97.88
5.00	22.00	5.00	0.70	8.00	6.00	101.46	97.65	100.55	97.708	103.44	99.81	102.44	97.71	99.74	99.10	102.42	99.51
7.00	24.00	4.00	0.60	12.00	6.00	99.55	99.28	98.93	103.21	102.34	99.98	102.20	99.28	97.19	100.50	98.95	101.11
6.00	24.00	5.00	0.40	10.00	2.00	99.58	98.68	102.26	99.54	101.77	97.88	99.38	99.12	102.44	102.36	101.28	97.59
8.00	24.00	1.00	0.70	4.00	4.00	100.68	95.36	100.43	98.71	99.84	99.20	101.56	100.01	98.76	98.77	99.39	100.63
4.00	22.00	1.00	0.60	10.00	5.00	101.46	98.59	98.35	100.62	101.43	98.78	97.78	100.47	101.24	100.75	98.42	98.73
7.00	18.00	1.00	0.50	8.00	2.00	99.45	98.45	96.42	99.43	100.08	99.13	98.49	101.45	98.90	100.39	99.72	101.97
Mean (%)						100.12	97.96	99.46	99.65	101.45	99.28	99.90	99.58	99.98	100.73	99.93	99.89
RSD (%)						0.98	1.84	1.85	1.82	1.46	1.12	1.89	1.34	1.89	1.40	1.32	1.71

(b) Prediction of validation set samples using ANN and MCR-ALS models																	
Concentration (µg/mL)						ANN						MCR-ALS					
PAR	HYO	PNP	PCA	TRO	PAP	PAR	HYO	PNP	PCA	TRO	PAP	PAR	HYO	PNP	PCA	TRO	PAP
8.00	18.00	5.00	0.60	6.00	3.00	100.03	99.84	99.25	99.12	100.53	101.86	98.21	102.14	100.88	101.91	98.68	101.20
6.00	18.00	2.00	0.70	12.00	5.00	100.26	100.08	99.24	98.56	99.69	99.57	97.82	100.22	98.37	99.25	99.12	98.78
5.00	22.00	5.00	0.70	8.00	6.00	100.70	100.09	100.19	97.92	99.78	100.16	100.23	100.64	97.93	99.54	99.02	100.81
7.00	24.00	4.00	0.60	12.00	6.00	101.34	99.91	101.75	98.87	99.72	99.76	101.87	100.41	101.44	101.56	100.91	101.08
6.00	24.00	5.00	0.40	10.00	2.00	100.64	103.61	101.04	98.73	97.99	98.33	101.71	100.49	102.19	98.83	98.47	99.91
8.00	24.00	1.00	0.70	4.00	4.00	100.56	99.87	103.27	99.00	100.09	99.62	98.70	99.50	97.17	103.01	98.733	99.16
4.00	22.00	1.00	0.60	10.00	5.00	98.01	97.26	101.27	100.88	100.02	97.23	99.61	101.04	99.82	97.78	97.73	102.23
7.00	18.00	1.00	0.50	8.00	2.00	99.96	99.86	98.10	102.62	100.05	100.44	99.28	98.91	98.77	102.76	101.66	101.02
Mean (%)						100.18	100.06	100.51	99.46	99.73	99.62	99.68	100.42	99.57	100.58	99.29	100.52
RSD (%)						0.98	1.71	1.64	1.53	0.75	1.38	1.51	0.96	1.80	1.95	1.32	1.14

**Table 3:** Performance parameters of the calibration and validation sets calculated for each proposed model; PLS and PCR (a) ANN and MCR-ALS (b)

(a) Performance parameters of the calibration and validation sets using PLS and PCR models												
Validation parameters	PLS						PCR					
	PAR	HYO	PNP	PCA	TRO	PAP	PAR	HYO	PNP	PCA	TRO	PAP
Linearity^a^	4.00–8.00	16.00–24.00	1.00–5.00	0.40–0.80	4.00–12.00	2.00–6.00	4.00–8.00	16.00–24.00	1.00–5.00	0.40–0.80	4.00–12.00	2.00–6.00
Correlation coefficient (r)^a^	0.9997	0.9995	0.9993	0.9993	0.9994	0.9995	0.9991	0.9991	0.9992	0.9996	0.9995	0.9993
Slope^a^	0.99336	0.99835	0.9711	0.97476	0.91544	1.0121	0.97322	0.9758	0.988	1.022	0.9404	0.9901
Intercept^a^	− 0.04762	− 0.02744	0.1031	0.001836	0.51118	− 0.06784	0.05264	0.4888	0.0542	− 0.02744	0.391	0.01216
RMSEC^b^	0.05	0.05	0.03	0.01	0.16	0.02	0.06	0.07	0.03	0.01	0.11	0.01
RMSEP^c^	0.07	0.07	0.05	0.01	0.24	0.04	0.10	0.10	0.04	0.04	0.23	0.07
LOD^d^	0.28	1.10	0.22	0.04	0.55	0.24	0.47	1.58	0.24	0.03	0.46	0.28
LOQ^d^	0.84	3.30	0.66	0.12	1.65	0.72	1.41	4.74	0.72	0.09	1.38	0.84

(b) Performance parameters of the calibration and validation sets using ANN and MCR-ALS models												
Validation parameters	ANN						MCR-ALS					
	PAR	HYO	PNP	PCA	TRO	PAP	PAR	HYO	PNP	PCA	TRO	PAP
Linearity^a^	4.00–8.00	16.00–24.00	1.00–5.00	0.40–0.80	4.00–12.00	2.00–6.00	4.00–8.00	16.00–24.00	1.00–5.00	0.40–0.80	4.00–12.00	2.00–6.00
Correlation coefficient (r)^a^	0.9993	0.9997	0.9998	0.9994	0.9998	0.9995	0.9998	0.9991	0.9992	0.9994	0.9998	0.9993
Slope^a^	0.9935	0.9608	0.9868	1.019	0.9749	0.9871	0.997	1.0008	0.993	1.028	0.9749	0.9871
Intercept^a^	− 0.0522	0.6488	0.0292	− 0.023	0.1146	0.034	− 0.0318	− 0.0512	− 0.019	− 0.0322	0.1146	0.008
RMSEC^b^	0.05	0.09	0.01	0.02	0.06	0.02	0.03	0.06	0.03	0.01	0.02	0.03
RMSEP^c^	0.08	0.14	0.02	0.01	0.09	0.03	0.04	0.09	0.05	0.10	0.09	0.05
LOD^d^	0.42	0.82	0.10	0.03	0.30	0.24	0.23	1.55	0.24	0.04	0.30	0.30
LOQ^d^	1.26	2.46	0.30	0.11	0.90	0.72	0.69	4.65	0.72	0.12	0.90	0.90

^a^Data of the straight line plotted between predicted concentrations of each component versus actual concentrations of the calibration set

^b^Root mean square error of calibration

^c^Root mean square error of prediction

^d^LOD and LOQ were calculated from the standard deviation (s) of the response and the slope of the calibration curve (S) according to the following equations: LOD = 3.3(s/S) and LOQ = 10(s/S)

### Greenness assessment

#### Green analytical procedure index

**Fig. 7:**
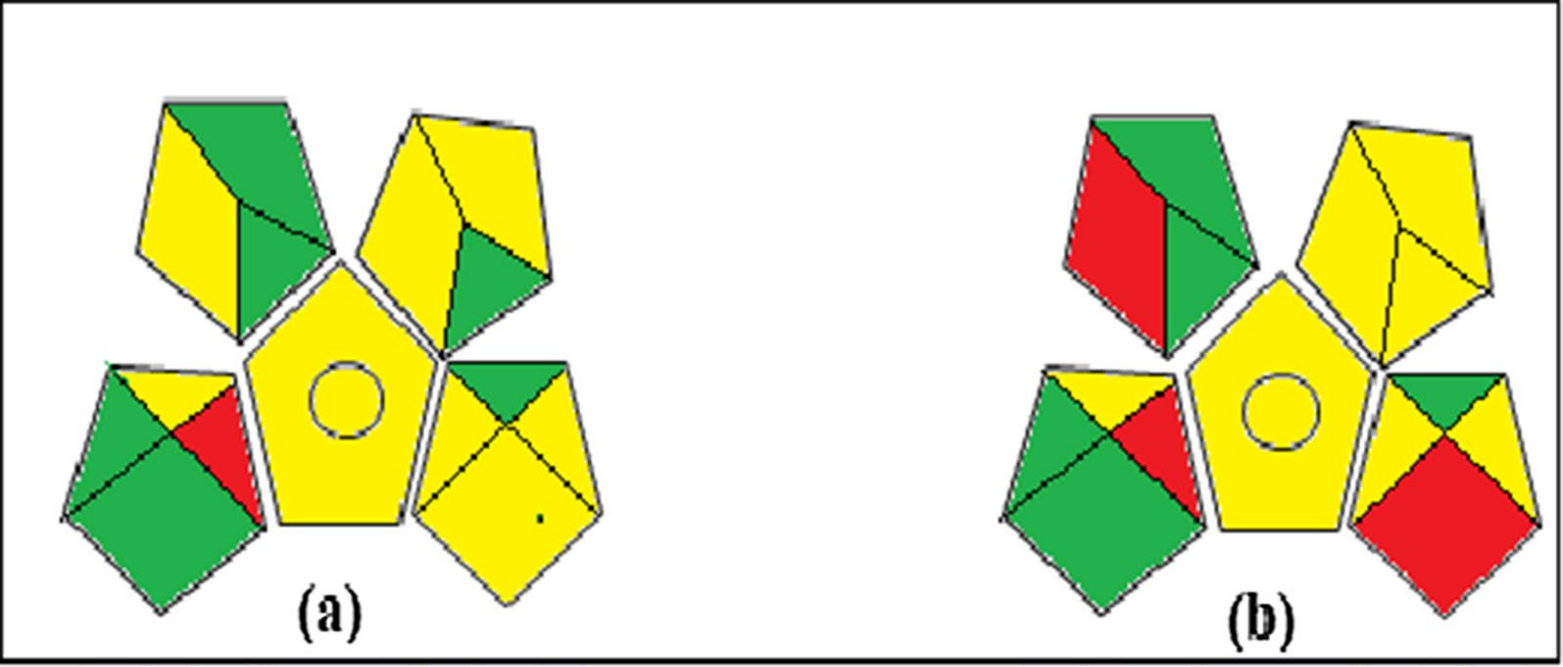
GAPI pictograms **a** for the proposed methods and **b** for reported method [29]

The GAPI tool assigned a green evaluation profile to the analytical processes it examined, as shown in (Fig. 7). As seen in (Fig. 7), the established techniques were straightforward, environmentally friendly, and capable of being used for both measurement and characterization without the need for extraction techniques as they have one red pentagram, while reported one has 3 red pentagrams. So, the established models were greener than the reported one. Additionally, they offered simple processes that generated the least amount of waste and dangerous substances.

#### Analytical GREEnness metric

**Fig. 8:**
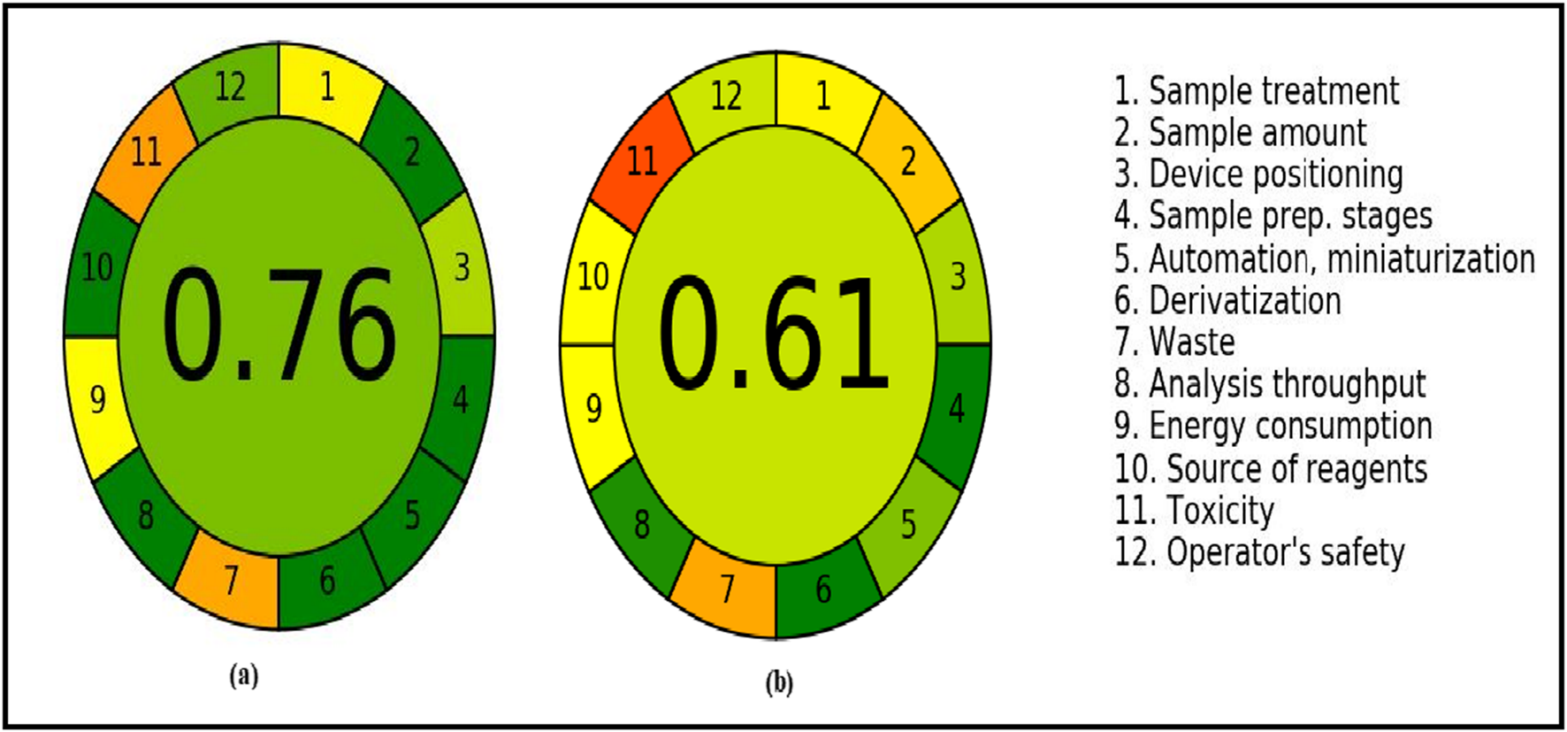
AGREE assessment of the green profile **a** for the evaluated methods and **b** for the reported method [29]. The total score is displayed in the center of the pictogram, with values near to 1 and dark green color representing that the evaluated method is more environmentally friendly

The colored pictograms in (Fig. 8) are shown for comparison between the suggested and the published techniques. The AGREE score for the established and reference methods was 0.76 and 0.61, respectively. So, according to score of AGREE the established methods mor green than the published one.

#### Analytical eco-scale

According to Table 4, the suggested methods result in 7 penalty points, which is ideal for green analysis because it produces less waste and potentially hazardous reagents while reported method penalty points was 12. After calculation of each penalty points for each analytical technique they are subtracted from 100 to calculate analytical eco-scale score. Analytical eco-scale scores of 100 indicate the optimal score for a green analytical method; scores of 75 or higher indicate excellent green analysis; scores of 50 to 75 indicate acceptable green analysis; and scores of less than 50 indicate insufficient green analysis.

**Table 4:** Penalty points (PPs) for proposed chemometric and reported HPLC methods

Parameters	Penalty points (PPs)	
	Proposed methods	Reported method [29]
Reagents		
Methanol	6.00	6.00
Water	–	0.00
Instrument		
Energy (˃ 0.1kWh per sample)	0.00	1.00
Occupational hazard	0.00	0.00
Waste	1.00	5.00
Total PPs	Ʃ7.00	Ʃ12.00
	93.00	88.00
Analytical Eco-scale score	Excellent green analysis	Excellent green analysis

Analytical eco-scale score = 100 (the ideal score of green analytical method)

Analytical eco-scale score > 75 (Excellent green analysis)

Analytical eco-scale score 50–75 (the green analysis is acceptable)

Analytical eco-scale score < 50 (the green analysis is inadequate

#### Comparative statistical study

PLS, PCR, ANN, and MCR-ALS models were used for the precise estimation of PAR and HYO in Buscopan Plus^®^ tablets. Statistics were used to compare the outcomes of the reported technique to those produced utilizing the existing regression procedures [29]. The F-ratio test and t-test revealed that the suggested techniques and the published one had an excellent agreement. The outcomes of the two tests did not differ much, and all information was compiled in (Table 5).

**Table 5:** Statistical analysis of the results obtained by the developed chemommetric methods and the reported HPLC methods for the determination of PAR and HYO in pharmaceutical preparation

Parameters	PAR					HYO				
	Proposed methods				Reported method^a^	Proposed methods				Reported method^a^
	PLS	PCR	ANN	MCR-ALS	HPLC	PLS	PCR	ANN	MCR-ALS	HPLC
Mean^b^ (%)	100.93	100.68	100.41	100.90	101.85	100.89	100.72	100.55	100.77	101.52
SD	0.86	0.75	0.72	0.77	0.48	0.85	0.65	0.88	0.83	0.52
Variance	0.74	0.56	0.51	0.60	0.23	0.73	0.42	0.78	0.69	0.27
N	6.00	6.00	6.00	6.00	6.00	6.00	6.00	6.00	6.00	6.00
Student’s t-teste (2.23)^c^	0.44	0.31	0.25	0.39	–	0.65	0.43	0.43	0.54	–
F-valuec (5.05)^c^	3.15	2.41	2.20	2.55	–	2.65	1.54	2.83	2.50	–

^a^HPLC method using water: methanol (50:50, V/V pH adjusted to 3.9 with CF3COOH acid) at flow rate of 1 ml/min, detection at 2210.0 nm [29]

^b^Average of 6 experiments

^c^Figures between parentheses represent the corresponding tabulated values of t and f at *P* = 0.05

The recovery% data were examined statistically using one way-ANOVA when the aforementioned methodologies were used to prepare medicines, but no appreciable differences were discovered (Table 6). These findings confirm the usefulness of the created models for accurate PAR and HYO calculation in pharmaceutical manufacturing.

**Table 6:** One-way ANOVA statistical analysis within 95% confidence interval on the recovery percentage data obtained from application of the chemometric methods and the reported HPLC methods on Buscopan^®^ plus tablet

	Source of variation	SS	*df*	MS	f	*P*-value	F-crit
PAR	Between groups	8.83	4	2.20	2.42	0.07	2.75
	Within groups	22.74	25	0.90	–	–	–
HYO	Between groups	3.29	4	0.82	1.55	0.21	2.75
	Within groups	13.22	25	0.52	–	–	–

**Fig. 9:**
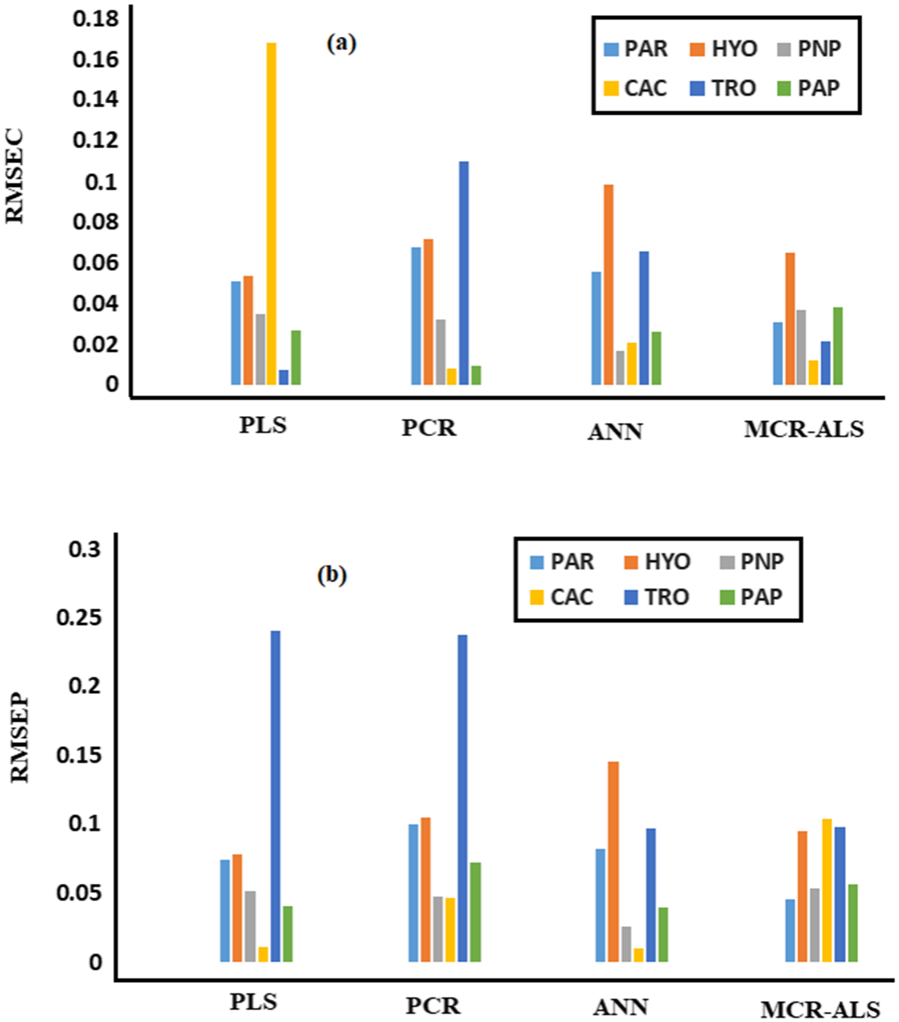
The calculated **a** RMSEC for each component obtained by the proposed validation models and **b** RMSEP obtained by the corresponding calibration model

For each component, column charts display the RMSEP and RMSEC calculated using the recommended validation and calibration models (Fig. 9). The MCR-ALS approach has the lowest RMSEP and RMSEC, per the findings. A diagnostic tool for evaluating prediction mistakes is RMSEP, indicating the method precision and accuracy. RMSEP evaluates the range of concentration changes and serves as a standard deviation [49]. The most effective model for selecting a numerical ingredient was determined to be the MCR-ALS method.

## Conclusion

The development of chemometrics offers numerous useful tools that make it easier to resolve complex spectrum data when studying drugs and their primary effects. The current study highlighted the importance of chemometry. It could be used with minimal modification and simple procedures for the simultaneous assessment of our binary mixture in dosage form. The tried-and-true green PLS, PCR, ANN, and MCR-ALS techniques were successfully used to evaluate the two drugs' and the contaminants' spectral profiles. It was found that the ANN model was the most accurate and precise model. Furthermore, the MCR-ALS technique is the only way to extract the spectral profiles of the six variables, and it is suitable for both qualitative and quantitative investigations. The present approach's greenness was considered in the early phases of development and then evaluated using the GAPI, AGREE index, and penalty point scoring system. The results showing that the developed models were greener than the reported one. The suggested chemometric techniques can be used for quality control analysis without the need for an initial separation step, and they are appropriate for determining the mentioned medications in their dosage form together with PAR impurities. When compared to the reported method, the methods are found to be equally sensitive with no discernible difference in precision. They can therefore be used in laboratories that do not have liquid chromatographic equipment. They could be used in the pharmaceutical formulation of the drug or for routine analysis of pure drugs.

### Supplementary Information

**Additional file 1. Fig. S1**: Chemical structure of (**a**) paracetamol, (**b**) hyoscine butylbromide, (**c**) p-aminophenol, (**d**) p-nitrophenol, (**e**) p-chloractanilide and (**f**) tropic acid.

## Data Availability

Data collected using a spectrophotometer and software. The corresponding author will provide the datasets created and/or analysed during the current study upon reasonable request.

## Abbreviations

PAR: Paracetamol
HYO: Hyoscine *N*-butyl bromide
PAP: P-aminophenol
PNP: P-nitrophenol
PCA: P-chloroacetanilide
TRO: DL-tropic acid
GAC: Green analytical chemistry
NEMI: National Environmental Approaches Index
AGP: Assessment of Green Profile
GAPI: Green Analytical Procedure Index
PLS: Partial least squares regression
PCR: Principal component regression
ANN: Artificial neural network
MCR-ALS: Multivariate Curve Resolution-Alternating Least Squares
HPLC: High performance liquid chromatography
LVs: Latent variables
RMSEC: Root mean square calibration error
FWHM: Full width at half maximum
MSE: Mean squared error
RMSEP: Root mean square error of prediction
μg: Microgram
mL: Milliliter
nm: Nanometer
ICH guidelines: International council on harmonization
SD: Standard deviation
RSD: Relative standard deviation
