# Supplementary material for: Development and validation of chemometric-assisted spectrophotometric models for efficient quantitation of a binary mixture of supportive treatments in COVID-19 in the presence of its toxic impurities: a comparative study for eco-friendly assessment
Source: BMC Chem. 2023 Dec 7;17(1):177. doi: 10.1186/s13065-023-01089-9 (PMC10701940; doi:10.1186/s13065-023-01089-9)
Supplement: Supplementary file 1 — Additional file 1. Fig. S1: Chemical structure of (a) paracetamol, (b) hyoscine butylbromide, (c) p-aminophenol, (d) p-nitrophenol, (e) p-chloractanilide and (f) tropic acid. [file 13065_2023_1089_MOESM1_ESM.docx]

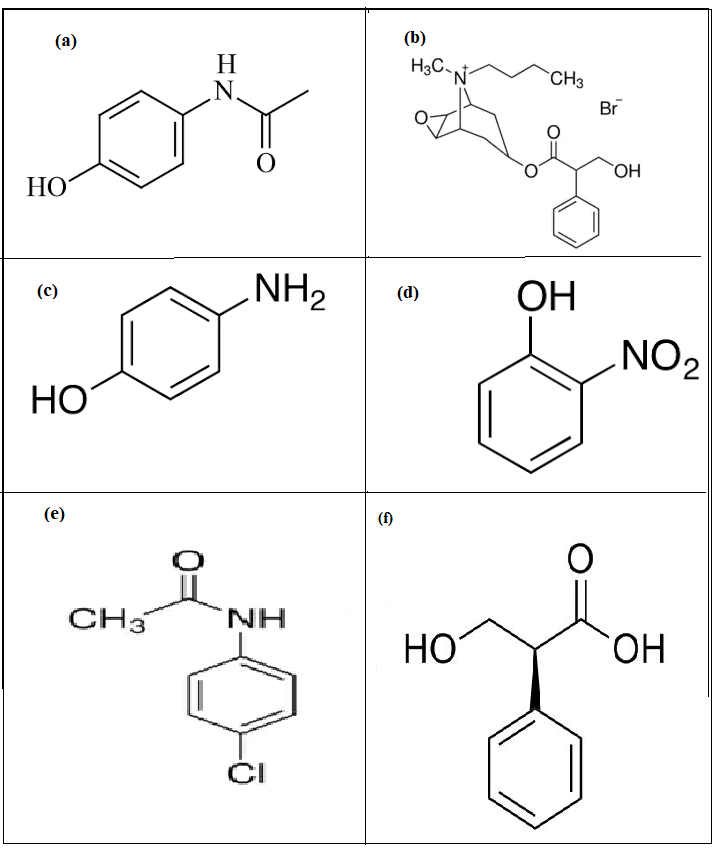


**Fig.1S**: Chemical structure of **(a)** paracetamol, **(b)** hyoscine butylbromide, **(c)** p- aminophenol, **(d)** p-nitrophenol ,**(e)** p-chloractanilide and **(f)** tropic acid
